# In Vitro Evaluation of Substantivity, Staining Potential, and Biofilm Reduction of Guava Leaf Extract Mouth Rinse in Combination with its Anti-Inflammatory Effect on Human Gingival Epithelial Keratinocytes

**DOI:** 10.3390/ma12233903

**Published:** 2019-11-26

**Authors:** J. Varghese, L. L. Ramenzoni, P. Shenoy, U. Y. Nayak, N. Nayak, T. Attin, P. R. Schmidlin

**Affiliations:** 1 Department of Periodontology, Manipal College of Dental Sciences, Manipal Academy of Higher Education, Tiger Circle Road, Madhav Nagar, Manipal, Karnataka 576104, India; jothimv@gmail.com (J.V.); namrata.nayak2328@gmail.com (N.N.); 2 Clinic of Conservative and Preventive Dentistry, Center of Dental Medicine, University of Zurich, Plattenstrasse 11, 8032 Zurich, Switzerland; thomas.attin@zzm.uzh.ch (T.A.); patrick.schmidlin@zzm.uzh.ch (P.R.S.); 3 Laboratory of Applied Periodontal and Peri-implantitis Sciences, Clinic of Conservative and Preventive Dentistry, Center of Dental Medicine, University of Zurich, Zurich, Switzerland, Plattenstrasse 11, 8032 Zurich, Switzerland; 4 Department of Microbiology, Kasturba Medical College, Manipal Academy of Higher Education, Tiger Circle Road, Madhav Nagar, Manipal, Karnataka 576104, India; padamaja.shenoy@manipal.edu; 5 Department of Pharmaceutics, Manipal College of Pharmaceutical Sciences, Manipal Academy of Higher Education, Circle Road, Madhav Nagar, Manipal, Karnataka 576104, India; usha.nayak@manipal.edu

**Keywords:** guava, human gingival keratinocyte, inflammation, biofilm, substantivity, staining

## Abstract

This study aimed to assess the biofilm reduction, staining potential, and cytotoxicity of guava extract mouth rinse compared to chlorhexidine (CHX). Substantivity, staining, and antibiofilm potential were investigated by spectrophotometry, colony-forming units, and luminosity color meter, respectively. The cell viability assay was conducted using a colorimetric assay to determine nontoxic levels of guava (0.15%) and CHX in human gingival epithelial keratinocytes (HGEK-16). Cells were treated with lipopolysaccharides (LPS, 1μg/mL) and guava to assess inflammatory gene expression levels of interleukin-β1, tumor necrosis factor-α, and Prostaglandin E2. A scratch wound healing assay investigated the effects of guava on cell migration. The teeth coated in guava mouth rinse displayed 19.4% higher substantivity compared to CHX (0.2%), and the anti-biofilm reduction was observed with both guava and CHX mouth rinses (P < 0.05). The overall discoloration changes were higher with CHX and distilled water compared to guava. Also, guava significantly enhanced HGEK-16 cell viability (P < 0.05), and IL-β1, TNFα and PGE2 expression presented a 0.6-fold decrease when exposed to guava and LPS (P < 0.05). The present study showed that guava mouth rinse fulfilled the requirement for an effective and useful oral care product with desirable substantivity and anti-biofilm action. In addition, guava reduced the inflammation response in HGEK-16 and may be a potential oral rinse for oral anti-inflammatory therapies.

## 1. Introduction

There has long been great interest both by dental professionals and commercial oral health care markets in remedial agents that could augment the mechanical elimination of biofilms present in the oral cavity. It has been well established that the pathogenic microbiota present in the biofilm strongly influences the development of periodontal disease and caries [1]. Hence, appropriate oral hygiene practices, along with professional oral prophylaxis, should be followed for effective oral care benefits. However, many patients are unable to reach acceptable levels of oral hygiene at home, especially when they have maligned teeth, prostheses, proximal restorations, orthodontic appliances, etc. [2]. Hence, plaque niches remain that may contribute to periodontal disease. Therefore, an adjunctive chemotherapeutic approach has been recommended, along with routine self-performed oral hygiene [3]. Among the commonly tested media, chlorhexidine (CHX) mouth rinse is still considered the gold standard for its ability to inhibit plaque growth and the resulting gingivitis. However, its effectiveness is dose-dependent, and hence the choice of dosage may vary, which can also affect levels of patient compliance. It is further known for its adverse side effects, namely staining and taste alteration, and hence it is not recommended for prolonged use [4]. A further shortcoming is its cytotoxicity on various cells, such as human gingival fibroblasts [5], periodontal ligament cells [6], and osteoblastic cells [7]. There are various clinical studies that have highlighted the antimicrobial effect of various chemical plaque control agents [3,8]. The efficacy of such agents is based on their substantivity, i.e., prolonged retention on the oral tissues [9]. This property can be evaluated by quantifying the reduction in salivary microbial counts subsequent to a single contact with the antimicrobial agent [10]. Another important factor that has been of utmost concern is the long-term use of synthetic chemical compounds that then facilitate bacterial resistance, thereby decreasing their usefulness [11]. Hence, the search continues for phytotherapeutic products, which provide similar clinical efficacy and, equally importantly, are cost-effective. A literature search supports the use of herbal products as an adjunctive mode of managing systemic and oral diseases [12,13,14].

*Psidium guajava* L. (commonly designated as “guava”) is a fruit tree belonging to the myrtle family (Myrtaceae). Guava has been successfully shown to treat a number of systemic ailments related to gastrointestinal disturbances, hormonal imbalance, mouth sores, bleeding gums, etc. [15]. The pharmacological basis for this may lie in various bioactive compounds present within the guava leaves (tannins, triterpenes, flavonoids, essential and fixed oils, saponin, carotenoids, lectins, vitamins, alkaloids, glycosides, quercetin, and reducing-sugars [16,17]. The presence of polyphenolic compounds, such as protocatechuic, ferulic, ascorbic, gallic, and caffeic acids, as well as flavonoids and quercetin accounts for the antibacterial and anti-oxidant effect, which could support the immune system and it has been positively considered as an adjuvant for treating periodontal disease [18]. Previous in vitro studies showed that guava leaf extract was found to reduce the expression of lipopolysaccharide-inducible pro-inflammatory mediators and is considered to be beneficial for biological cell activity [19]. However, there is a lack of scientific data regarding cell cytotoxicity of the guava leaf on oral gingival cells and its effect on inflammation caused by oral bacterial lipopolysaccharides (LPS). Moreover, there is as yet insufficient knowledge on how guava leaf extract may influence oral biofilm formation, which could clarify its usefulness as a mouth rinse in oral therapies.

Thus, this in vitro investigation explored the characteristic properties of a mouth rinse containing guava extract and compared it to the standard 0.2% CHX mouth rinse as a control. The two null hypotheses tested were that, firstly, guava extract mouth rinse would not be as effective as 0.2% CHX in reducing biofilm formation and, secondly, it would cause staining on enamel, dentin and composite restorative material similar in extent to that produced by the standard CHX mouth rinse. The substantivity of the experimental mouth rinse was studied first, followed by measurements of its antimicrobial effect and staining properties. In addition, this study evaluated the cytotoxicity, anti-inflammatory activity, and wound healing potential effect of guava on human gingival epithelial keratinocytes. 

## 2. Materials and Methods

The present study was approved by the Institutional Ethics Review Board (IEC 43/2018) and was conducted in accordance with the Helsinki declaration of 1975, as revised in 2000.

### 2.1. Plant Materials 

*Psidium guajava* leaves were collected from the university campus during the months of June and July. The leaves of the guava plant were identified and authenticated by the Department of Botany & Taxonomy, Karnataka, India. A voucher specimen (specimen no. PP620) was deposited in the Department of Pharmacognosy, Manipal College of Pharmaceutical Sciences, Manipal Academy of Higher Education, Manipal, Karnataka, India.

### 2.2. Preparation of the Guava Mouth Rinse 

After harvesting, the *Psidium guajava* leaves were thoroughly cleaned, shade-dried, and finely powdered. The guava leaves were ground to a coarse powder. The cold extraction or maceration procedure was utilized for the preparation of hydro-ethanolic guava extract. The powder was placed into a stoppered container with hydro-ethanol (70% water and 30% alcohol) for seven days, with frequent agitation, then filtered, and the marc was pressed. The solvent was then carefully evaporated in a vacuum evaporator (Rotavapor R-100, Buchi, Mumbai, India) to obtain 50 g of dry extract. Next, the formulated extract was checked on various dilutions of hydro-ethanolic extract. All the concentrations were tested against commonly occurring oral bacterial strains (*Streptococcus mutans, Streptococcus oralis, Streptococcus mitis, Actinomyces viscosus, Fusobacterium nucleatum*). The minimum inhibitory concentration (MIC) value of the extract was determined as the lowest concentration that completely inhibited bacterial growth after 48 h of incubation at 37 °C. Based on the MIC result, an average value of 0.15% was taken for the formulation of the mouth rinse by dissolving guava leaf extract (0.15%) in absolute alcohol (1.1%) and homogenizing it with glycerol (15%), propylene glycol (15%), tween 80 (1%) and menthol (0.1%). The prepared mouth rinse was subsequently used as the experimental agent for further microbiological and cell studies.

### 2.3. Assessment of Substantivity 

The current research followed the methodology adopted in an earlier study [20]. Twenty human periodontally healthy premolar teeth (n = 10 per group) extracted for orthodontic purposes were collected and segmented with a water-cooled diamond saw to retrieve only the crowns. The fragments were inserted in polystyrene resin (Antares Chem Private Ltd., GE, Mumbai, India), leaving the vestibular surfaces exposed. Thereafter, the resin-coated surfaces of the crowns were coated with nail paint and randomly allotted to assess substantivity, by 1 min immersion into either group 1—0.15% guava mouth rinse or group 2—0.2% CHX mouth rinse gluconate (Periogard-Colgate). The time intervals for the study were 5 min, 30 min, and 360 min. The specimens were immersed in 1 mL of distilled water and kept in glass tubes. After the specified time intervals, an aliquot was taken from the tubes, and the same volume was immediately replaced and analyzed by UV spectrophotometer at 260 nm for chlorhexidine and at 274 nm for guava (Table 1).

### 2.4. Biofilm Development 

The methodology used for biofilm development was taken from a previous study [21]. Biofilms included the following bacterial species: *Streptococcus mitis* (ATCC^®^ 49456™), *Streptococcus oralis* (ATCC^®^ 35037™), *Streptococcus mutans* (ATCC^®^ 25175™), *Fusobacterium nucleatum* (ATCC^®^ 25586™) and *Prevotella intermedia* (ATCC^®^15033™). A total of 30 samples (n = 10 for each of three groups) of human teeth extracted for the purpose of orthodontic treatment were used for the present work. Following extraction, the teeth were thoroughly debrided and instrumented to eliminate adherent soft tissues and calculus. The teeth were then disinfected and cleaned with a 2.5% Sodium hypochlorite solution. The teeth were decoronated and split longitudinally. The resultant crown fragments were designed in a rectangular shape (approximate dimensions of 5 mm × 2 mm × 1 mm) using a low speed diamond point (Horrico, Berlin, Germany). The tooth specimens were placed in 24-well polystyrene cell culture plates which were covered with saliva (800 µL) and a modified fluid universal medium (800 µL mFUM). A 67 mmol^−1^ Sørensen’s buffer solution (38% *v*/*v* pH 7.0) containing 0.15% *w*/*v* glucose + 0.15% sucrose was added to this mixture. The wells were inoculated anaerobically at 37 °C with 200 µL of the above-mentioned microbial species suspension prepared from a concentration of each of these microbial species for 0.5 h, 16.5 h, 40.5 h, and 64.5 h. The medium was replenished by aspiration of the expended medium from the wells and the use of fresh medium after 16.5 h, 24 h, and further at 40.5 h interval (Table 2).

### 2.5. Assessment of the Anti-Biofilm Effect

The tooth specimens coated with the cultured biofilm were submerged for 1 min in 1 mL of the test agents (0.15% guava mouth rinse/0.2% CHX mouth rinse/distilled water) and then rinsed by dipping three times in 2 mL of physiological saline solution. The tooth specimens coated with biofilms (n = 10 per group) were exposed to the test agents for 16.5 h, 20.5 h, 24.5 h, 40.5 h, and 48.5 h [21]. At the end of the final treatment, the biofilms were left undisturbed and later harvested after 64.5 h by vortexing using 1mL physiological saline. Further, aliquots of cultured biofilm were sonified, diluted, and placed onto a Columbia agar base (Criterion™, Santa Maria, CA, USA) containing 5% *v*/*v* sheep blood and incubated anaerobically at 37 °C. Thereafter, colony-forming units (CFU) were counted 72 h after plating with the aid of the EC2™ automated colony counter (BioMérieux Inc, Hazelwood, MO, USA).

### 2.6. Staining Potential 

The in vitro method of reproducing stain was carried out as previously proposed [22]. In order to prepare the standard tea solution (Marks and Spencer extra-strong loose tea, Chester, UK), 8 g of tea leaves were boiled in 800 mL of water for 2 min. The tea solution was then refrigerated at 4 °C for half an hour, and thereafter, filtered to remove the tea residues. Then the tea solution was kept at room temperature during the course of the study. This experiment involved the use of extracted human teeth (total = 30) and restorative material (total = 15) (Table 3). The tooth specimens were mechanically sectioned. Both the enamel (n = 15) and dentin (n = 15) aspects were utilized for the three groups involved in the study. For dentinal exposure, the labial surface of the crown was prepared using a diamond point (Horrico, Berlin, Germany). The specimens were then smoothened using a silicon carbide disc (600 grit) to obtain a flat surface, and finally polished with 0.1 µm alumina suspension (Ultra-Sol R; Eminess Tec Inc, Monroe, NC, USA) on a rotary felt disk. The restorative materials (n = 15) were prepared in Teflon molds (10 mm oval, 3 mm thick). Then, they were placed incrementally and cured with a UV light (Ivoclar Vivadent AG, FL-9494, Schaan, Liechtenstein). Curing cycles were performed at 40 s, 60 s, and finally for 5 min. Subsequently, the restorative specimens were embedded in visibly clear epoxy resin (Dentsply Sirona, York, PA, USA) which was mixed as per the manufacturer’s instructions. The dimensions of the restorative samples measured 15 mm in diameter to fit the optical lens of the i1Pro spectrophotometer (X-Rite, Grand Rapids, MI, USA). The restorative specimens were divided into three groups of five specimens each. For this experiment, initially, saliva was collected from healthy volunteers in the early hours from 8 am–11 am, whereby the patients had not consumed food for at least 2 h beforehand. The following methodology for staining by exposure to the experimental agents was applied, with the whole cycle being repeated six times during the course of 11 h. All the specimens were immersed in human saliva for 2 min at 37 °C. They were then rinsed with 2 mL of distilled water four times. Thereafter, specimens in each group were immersed into the experimental agents or the distilled water negative control for 2 min. Subsequently, specimens were washed consecutively four times with 2 mL of distilled water and then bathed in a standard tea solution (prepared earlier) for at least an hour. The final rinse involved four washes with 2 mL of distilled water followed by drying the specimens with compressed air for measurement of luminosity using the CIELAB (L*a*b*) color meter.

### 2.7. Assessment of Stains on Specimens

Prior to the commencement of the study, baseline L*a*b* measurements of the specimens were noted using the X-Rite, i1Pro spectrophotometer (holographic diffraction grating with 128-pixel diode array, wavelength range 380–730nm). The post-treatment readings of change in luminosity (L*), red-green axis (a*), yellow-blue axis(b*) were also documented.

The overall color changes were calculated as

∆E*ab = [(∆L*)2 + (∆a*) + (∆b*)2]1/2

In which,

∆L* = L* post − L *pre

∆a* = a* post − a*pre

∆b* = b*post − b*pre

### 2.8. Cell Viability Assay

Human gingival epithelial keratinocytes (HGEK-16) cell viability was determined by a 3-(4,5-dimethyl-2-thiazolyl)-2,5-diphenyl-2H-tetrazolium bromide (MTT, Sigma–Aldrich) dye reduction assay (5 mg/mL in phosphate-buffered saline). HGEK-16 cells (1 × 10^5^ cells/mL) were re-cultured in six-well plates supplemented with a fresh medium containing guava at a final concentration of 0.15%. After an exposure time of 24 h, the solutions were aspirated, and the cells washed with 1× phosphate-buffered saline (PBS) before the culture medium was added anew. At 48 h after exposure to the respective guava solutions, 500 mL of MTT was added to each well and incubated for 4 h at 37 °C in the dark. In the next step, MTT was removed by aspiration from the wells, and isopropanol was added (200 mL, 1N HCl) to solubilize the MTT-formazan crystals which had formed. The absorbance was measured at a wavelength of 570 nm with a spectrophotometer reader. All samples were tested in triplicates, and three independent experiments were performed.

### 2.9. Cell Culture and LPS-Induction

Immortalized human gingival epithelial keratinocytes (HGEK-16) were donated by the Oral Microbiology Institute, Center of Dental Medicine, University of Zurich. HGEK-16 cells were cultured in an incubator (5% CO_2_, 95% air humidity at 37 °C) and passaged at regular intervals depending on their growth characteristics using 0.25% trypsin (Seromond Biochrom, Berlin, Germany) and maintained in a complete epithelial medium (CM-DKSFM) consisting of a defined keratinocyte serum-free medium (Gibco, 10744-019), supplemented with 100 U/mL penicillin (Sigma, 15140-122), 100 mg/mL streptomycin (Sigma, 15140-122), 2 mM L-glutamine (Sigma, G7513), and 0.25 mg/mL fungizone (Sigma, 15290-018). The medium was changed every three to four days, and cells passaged once a week. The cells used in this study were between the fifth and fifteenth passage. There were four treatment groups used for this study: 1) positive control, HGEK-16 with induced inflammation using 1μg/mL lipopolysaccharide (LPS from *Porphyromonas gingivalis*, InvivoGen, San Diego, CA, USA); 2) negative control, HGEK-16 without any treatment; 3) test group 1, HGEK-16 cells with 1 μg/mL LPS and 0.15% guava added; and 4) test group 2, HGEK-16 cells with only 0.15% guava added. The cells were incubated for 24 h, and cell-free supernatant was used for the *interleukin-β1* (*ILβ1*), *tumor necrosis factor-α* (*TNFα*), and prostaglandin E2 (*PGE2*) gene expression assay.

### 2.10. Real-Time Quantitative Polymerase Chain Reaction Analysis (RT-PCR) 

The anti-inflammatory effect of the guava extract mouth rinse was tested by analyzing gene expression of markers of inflammation *ILβ1, TNFα* and *PGE2*. First, total RNA from the cells was isolated using trizol (Invitrogen, Grand Island, NY, USA) TRIZOL reagent and RNAeasy Mini kit (QIAGEN), 24 h after exposure to guava extract mouth rinse. Primers for inflammatory genes encoding *ILβ1, TNFα,* and *PGE2* were designed from Primer3 (version 0.4.0) as follows: *ILβ1* (forward primer: 5′-TAG AGC TGC TGG CCT TGT TA-3′, reverse primer: 5′- ACC TGT AAA GGC TTC TCG GA-3′, *TNFα* (forward primer: 5′-TGC CTA TGT CTC AGC CTC TT-3′, reverse primer: 5′-GAG GCC ATT TGG GAA CTT CT-3′), *PGE2* (forward primer: 5′-ATG GGC AAT GCC TCC AAT GAC TCC-3′, reverse primer: 5′-GCA CGC GCG GCT CTC GGG CGC CAG-3′) and *GAPDH* (forward primer: 5′-GCT CTC TGC TCC TCC CTG TT-3′, reverse primer: 5′-CAC ACC GAC CTT CAC CAT CT-3′). Following trizol extraction, real-time quantitative Polymerase chain reaction as performed using 15 mL final reaction volume of SYBR Green real-time master mix kit (Applied Biosystems, Grand Island, NY, USA). Total RNA (40 ng) was used per sample well. Each sample contained pooled mRNA from trizol extractions collected from the cell cultures exposed with and without LPS + 0.15% Guava at 24 h. All samples were tested in triplicates, and three independent experiments were performed. The 2^−∆∆ct^ method was used to calculate gene expression levels relative to *Glyceraldehyde 3-phosphate dehydrogenase* and normalized to negative control cells.

### 2.11. Scratch Wound Healing Assay (Cell Migration)

To determine the effect of guava on wound healing, a scratch-wounded HGEK-16 monolayer model was employed. The cells (1 × 10^5^ cells/mL) were plated onto a six-well plate and cultured under serum starvation to a maximum of 60% of confluence. Prior to the scratch assay, the cells were exposed to 10 μg/mL of mitomycin C (Sigma-Aldrich) in serum-free media for 2 h, which inhibited mitosis of the cells. Further, the scratch was also produced 16 h after the beginning of serum starvation of the cells, to halt proliferative response during wound closure. Next, each well was wounded by scratching with a 10 μL pipette tip. Following 1x PBS washes to remove cell debris, the cultures were exposed to 1 mL of: 1) 1 μg/mL LPS (positive control); 2) 1× PBS (negative control); 3) 1 μg/mL LPS and 0.15% guava; and 4) only 0.15% guava. Cell counting was also performed using a hemocytometer (Sigma Aldrich, St. Louis, Missouri, USA) before and after the closure to assure a low change in cell numbers between the start and end of the experiment. Digital images were captured using a camera-equipped, inverted microscope (Carl Zeiss, Inc., Thornwood, NY, USA), and wound width measurements were subtracted from wound width at time zero to obtain the net wound closure. The distance between edges of the injured monolayer was measured by Image J software (Software 1.48q, Rayne Rasband, National Institutes of Health, Bethesda, MD, USA) (NIH) in pixels and wound closure was expressed as the difference in width at 0 h, 12 h, and 24 h after wound simulation. Since the scratch width varied to some extent from one wound to another, a “relative wound closure” (RWC) area was calculated by normalizing the measured wound closure area (in pixels) to the total area of the image, which is covered in pixels (RWC (%) = wound closure area (pixel) × 100 (%)/× (pixel)).

### 2.12. Statistical Analysis

For the substantivity and antibiofilm assessment, the data pertaining to the addition of different experimental agents to the total biofilm population were analyzed using log 5-transformation. The skewed distribution of the data was analyzed and required nonparametric statistical tests. Hence, comparisons within the experimental groups were performed using the Kruskal–Wallis test. For staining potential, the comparison of the mean color changes in various experimental products used in the study pre and post-treatment was analyzed by using the Mann-Whitney post hoc test. For the HGEK-16 cell culture experiments, an analysis of variance (ANOVA) was used for significant differences in results followed by the post hoc Fisher least significant difference (LSD) test. The paired two-tailed t-test was used to compare individual groups with each other. All pairwise comparisons yielded significant differences according to Tukey’s test. Statistical significance was set at P < 0.05. Statistical analysis was performed using the statistical software package SPSS 22.0 software for Windows, and the values are shown with median and IQR from three different experiments in the case of nonparametric tests, each one performed in triplicate.

## 3. Results

### 3.1. Substantivity Property

The results related to the substantivity of the guava extract mouth rinse are summarized in Table 1. Group 1 samples, which were soaked in 0.15% guava mouth rinse, showed desorption of 8.14 µg/mL at 5 min and exhibited a gradual decrease in the concentration to 5.87 µg/mL at the end of 360 min. In contrast, Group 2 samples soaked in 0.2% CHX mouth rinse showed peak desorption of 177.24 µg/mL at 5 min, which reduced to a concentration of 124.82 µg/mL at 360 min. On completion of 360 min, the concentration in group 1 was 19.4%, while in group 2, it was 10.25%.

### 3.2. Anti-Biofilm Effects

The anti-biofilm effects are summarized in Table 2. At baseline, the initial microbial count on all tooth specimens was standardized to five log steps. Among all the groups tested for antimicrobial effect, the distilled water group displayed the highest number of microorganisms (1.23 × 10^9^ ± 2.24 × 10^11^). The addition of 0.15% guava extract reduced the biofilm growth by 4 log steps (1.5 × 10^1^ ± 0.15 × 10^1^, P < 0.05) at the end of 72 h. Similar results were obtained from the 0.2% CHX mouth rinse, which exhibited almost complete inhibition (0.5 × 10^1^ ± 0.1 × 10^1^; P < 0.05).

### 3.3. Staining Potential

The results are summarized in Table 3. All tooth specimens exhibited stains developed by the test agents, which turned to be darker over the course of time. The darkening (∆L*) and discoloration (∆E*) formed on enamel specimens after staining with test agents were found to be statistically non-significant; whereas on the dentine specimens (∆L*) and (∆E*) presented statistical changes in all the three groups, with maximum staining effect displayed by the CHX group. However, there was no statistical significance in the staining caused by the guava mouth rinse and distilled water. The composite substrates exposed to the test agents also revealed a statistical significance in (∆L*) and (∆E*) among the distilled water and CHX group, wherein distilled water group exhibited more staining compared to CHX. The change along the red-green axis (∆a*) showed more significance after exposure to all test agents. All the three specimens displayed a statistically significant difference in ∆a*. In the enamel specimens, both guava and CHX mouth rinse groups exhibited statistically significant changes compared with the distilled water group. Considering ∆a* variations in both dentine and composite substrates, a statistically significant difference was observed among all the three test groups, in which the CHX group posed obvious staining.

### 3.4. Effect of Guava Mouth rinse on HGEK-16 Cell Viability

**Figure 1:**
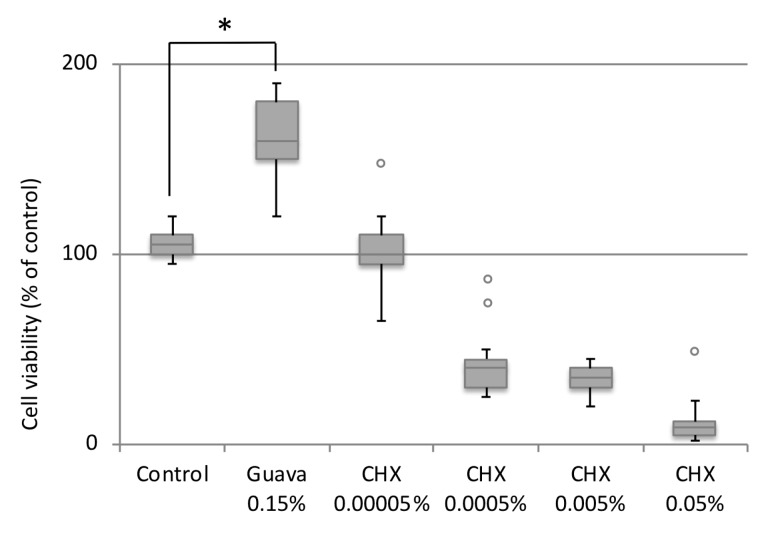
Evaluation of the cellular viability using cell viability assay of human gingival epithelial keratinocytes. A significant increase in cellular viability was detected for guava treatment (0.15%), whereas, CHX at concentrations higher than 0.00005%, were found to be cytotoxic. X-axis = points of measurement, Y-axis = optical density, * P < 0.05. Mean ± S.D.

The MTT cell viability or cytotoxicity assays showed significantly enhanced HGEK-16 cell viability after 24 h guava mouth rinse exposure compared with the untreated control cells (P < 0.05 at 24 h). Guava showed a significant increase of ~50% compared to control (Figure 1). Based on these results and following the microbiological experiments, the concentration of 0.15% guava was selected for further gene expression and cell migration analysis. Regarding CHX treatments, the cell viability was decreased when HEGK-16 cells were treated with CHX at concentrations higher than 0.00005% (Figure 1).

### 3.5. Anti-Inflammatory Potential of Guava Mouth rinse

**Figure 2:**
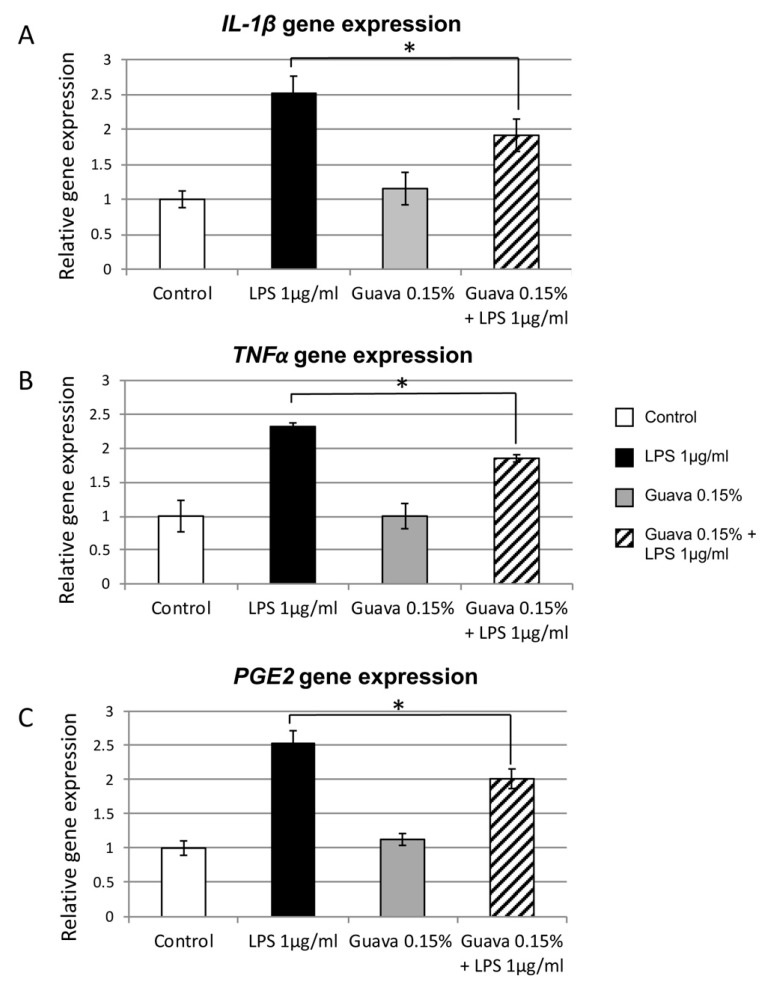
Upregulation of inflammatory genes in HGEK-16. Increased expression of (**A**) *ILβ1*, (**B**) *TNFα*, and (**C**) *PGE2* genes was found in LPS (1 μg/mL) compared to control (white bars). A reduction of expression was found on HGEK-16 upon treatment with guava 0.15% mouth rinse and LPS (1 μg/mL). x-Axis: LPS and guava treatments; y-Axis: relative gene expression. * P < 0.05. Mean ± S.D.

To investigate whether a guava extract mouth rinse could inhibit LPS-induced inflammatory gene expression, HGEK-16 cells were pretreated for 24 h with: (1) 1 μg/mL LPS (positive control); (2) 1× PBS (negative control); (3) 1 μg/mL LPS and 0.15% guava; and (4) only 0.15% guava. The RT-PCR results gene expression results showed that LPS induction treatment alone increased gene expression up to 2.5-fold of ILβ1, TNFα, and PGE2 compared with the negative untreated control (Figure 2A–C, P < 0.05). Thus, the positive control LPS-induced HGEK-16 without treatments showed the highest upregulation for all pro-inflammatory cytokines tested in this study. The LPS treatment as a positive control was used as a standard to obtain guava inhibition activity toward ILβ1, TNFα, and PGE2. When cells were simultaneously exposed to guava and LPS, there was a ~0.6-fold decreased of ILβ1, TNFα and PGE2 pro-inflammatory marker expression compared to the LPS positive control treatment (Figure 2A–C, P < 0.05).

### 3.6. Scratch Wound Healing Assay (Cell Migration)

**Figure 3:**
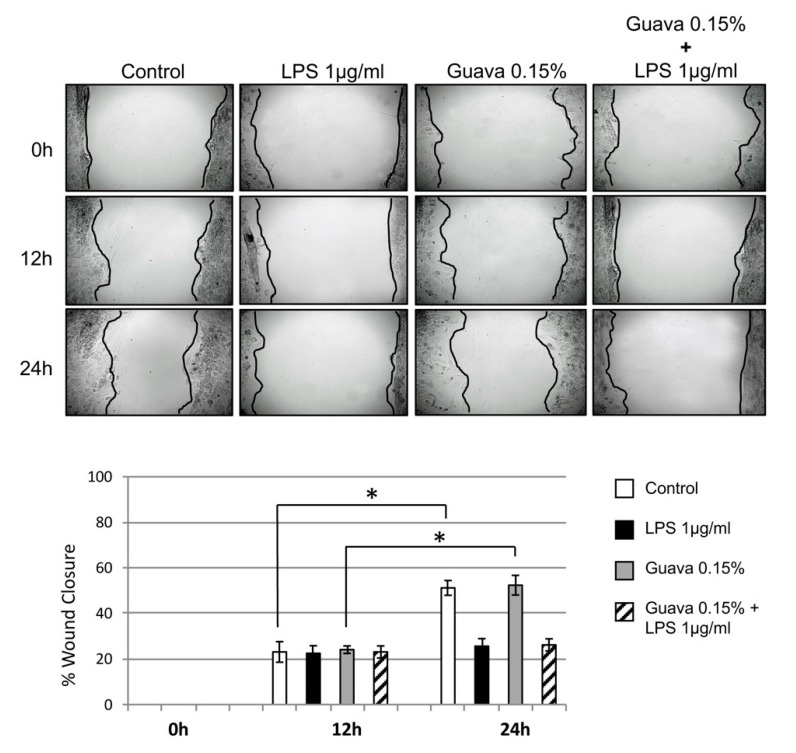
Effects of guava exposure on wound healing of confluent HGEK-16. During the 12–24 h after wounding, a significant wound closure rate was found to be similar for guava (0.15%) and the untreated control. No significant migration difference was found after guava (0.15%) and LPS (1 μg/mL) treatment. Each bar indicates the mean ± S.D. Wound closure rates are expressed as the difference between wound width at 0 h, 12 h, and 24 h (n = 3). * P < 0.05.

Cells exposed to guava extract mouth rinse at concentrations of 0.15% showed significantly higher wound closure rates at 24 h as compared to untreated control cells (Figure 3, P < 0.05). However, over the same time period, wound closure was not significantly increased with exposure to guava + LPS, and migration was similar to the LPS cell-treated group (P < 0.05). Wound closure was 60% complete at 24 h for both control and guava treated groups, whereas it was 20% complete in the LPS cell treated group and the guava + LPS treated group (Figure 3).

## 4. Discussion

The present study evaluated the biofilm reduction potential and staining properties of a herbal mouth rinse formulation containing guava extract and compared them to CHX mouth rinse as a commonly prescribed standard chemoprophylactic anti-biofilm agent. The cytotoxicity, anti-inflammatory effect, and wound healing activity of the guava extract mouth rinse on gingival epithelial keratinocytes was also evaluated. The substantivity of test agents is the key feature for providing antimicrobial efficacy. Previous studies performed have stated that the prolonged action of chlorhexidine within the oral cavity can be attributed to its adsorption on surfaces, which in turn plays a critical role in plaque inhibition [9]. This inhibitory effect resulted in a measurable reduction in the bacterial count after a single exposure to the antimicrobial agent. As tested in this study, both experimental agents showed substantivity on the tooth specimens, even at the end of 360 min (Table 1). Earlier studies have found that a single dose of CHX mouth rinse resulted in measurable antibacterial activity for 5 h or more [9,23]. Hence, this retentive feature of the guava extract mouth rinse could also enhance its antimicrobial effect. At the specified study time intervals, a 0.2% CHX gold standard chemotherapeutic agent presented almost complete inhibition of microbial growth, which resulted in confirmation of our first null hypothesis. However, the guava extract mouth rinse also exhibited a beneficial reduction in bacterial counts compared to the distilled water group (Table 2). This favorable outcome could be attributed to the presence of its pharmacologically active components (flavonoids, guavanoic acid, guajaverin, and guayavolic acid) present in the guava leaf [17]. Likewise, in a separate study, a similar effect was noticed against *Streptococcus mutans* biofilm using guava leaf extract [24]. In a further study, experiments on inhibition, adhesion, and co-aggregation of plaque pathogens demonstrated the antiplaque activity of guava extract, and it was concluded that guava has antiplaque action and aids in the maintenance of the resident microflora [25]. The antibacterial efficacy of hydro-ethanolic guava leaf extract was also assessed against *Lactobacillus acidophilus*, with the results showing it to be as efficacious as 0.2% CHX. The substrates in the present study were selected to mimic the oral environment in clinical settings. Dentin exhibited more pronounced staining when exposed to the test agents than did enamel and composite restorative material. A literature review pertaining to the overall color change (∆E*) showed that values greater than 3.3 were considered visually undesirable [26,27,28,29]. The findings of the current study show that overall color change (∆E*) with distilled water and guava mouth rinse were comparatively less staining than the 0.2% CHX rinse (Table 3). Through the findings of numerous studies, it is universally accepted the knowledge that CHX mouth rinses cause extrinsic staining [4,30]. Thus, our findings lend further support to the fact that the 0.2% CHX mouth rinse stains more profusely on all substrates, resulting in a rejection of our second null hypothesis. An interesting finding in the present study was that the distilled water group exhibited a clinically undesirable color change (∆E*) on all substrates. This could be attributed to the fact that saliva and tea alone can result in staining. This finding was contrary to the results of other studies that found significantly less staining when specimens were subjected to submersion protocol using saliva, water, tea, and CHX [22,31]. Another notable observation in this study was that the composite restorative material showed overall less discoloration (∆E*) and change in luminosity (∆L*) compared to enamel and dentin, which is consistent with a previous study [32]. Further, among the three test agents used in this study, guava mouth rinse stained least on composite restorative material compared to CHX and distilled water. Possible explanations could be the mild concentration of guava mouth rinse used in the study, the mechanism of its binding to the restorative material, or that the guava mouth rinse simply neutralized the discoloring components of the tea on the composite material. Further research is needed to clarify this issue. When considering the methodology employed in staining studies, previous studies have found that substrates pre-treated with saliva formed acquired pellicle which resulted in reduced CHX and tea staining. However, a pellicle may inversely assist in the binding of either tea or CHX alone to hydroxyapatite [33]. Authors in another study pointed out that there was no particular agreement on a correct methodology to test the staining potential on substrates, which may lead to inconclusive results [32].

Another purpose of this study was to determine the cell viability, anti-inflammatory, and wound healing effect of guava extract mouth rinse on human gingival epithelial keratinocytes, which were also inflamed after exposure to LPS. Our results suggested that guava extract mouth rinse at a concentration of 0.15% had a positive effect on oral gingival keratinocyte cell viability compared to the untreated control (Figure 1). However, previous studies conducted on in vitro cell cultures treated with different extracts of the guava leaves showed that ethanol leaf extracts tend to have a cytotoxic effect at high concentrations [34]. Moreover, hot-water guava extract has been shown to have a greater effect and lower cytotoxicity [35]. Consequently, the concentration of guava extraction in the test solution should be carefully controlled for in vitro assay applications. The concentration of 0.15% guava extract, shown to be safe based on the cell viability test, was further used for the anti- potential inflammatory analysis. In the current study, guava mouth rinse presented a reduction of expression of inflammatory markers (Figure 2A–C). *IL-β1, TNFα,* and *PGE2* were decreased in gene expression when cells were exposed to the combination of guava and LPS (Figure 2). Our findings corroborate previous studies that confirmed guava extract to be a powerful anti-inflammatory agent [19,36,37]. The inflammation inhibitory effect was found to be closely linked to the phenolic and flavonoid content of the guava extract [38,39]. Moreover, inhibition of LPS-induced production of pro-inflammatory mediators was due to the ability of the guava leaf extract to suppress phosphorylation in inflammatory protein expression [40]. What is interesting to note is that this inflammatory inhibition was improved after the optimization of extraction conditions of the guava leaves [40]. Methanol and ethanol guava leaf extracts have shown to be potent immune-stimulant agents [41]. However, it is important to consider that differences reported in cell trial results reported were due to differences in extraction methods and to the doses assayed, or even in variations in the harvesting time of the leaves. In this study, we have utilized hydro-ethanolic guava extract, which was reported to possess significant antioxidant, anti-tumor, and anti-inflammatory activity [42].

Regarding the possible wound healing effect of guava, our in vitro scratch wound healing assay found only non-significant differences to the control (Figure 3). This, despite the fact, that guava’s wound healing properties have been reported favorably in earlier pharmacological animal studies [43]. Furthermore, cancer cell in vitro studies have shown that guava extracts have a significant anti-migration and anti-angiogenesis activity [34], while this effect is not found in normal cells. The unchanged migration could be explained by the lower concentration of guava extract used, but this point deserves further examination on a molecular basis.

This study has demonstrated that ethanol leaf extract has a desirable cell viability effect on the HEGK-16. The positive outcome also involved anti-inflammatory action, which demonstrates the importance of the in vitro approach to evaluate cell biological response to guava extracts. Additionally, the study showed the advantage of guava mouth rinse use as it contributes to biofilm reduction with less discoloration compared to CHX. However, the findings obtained here were performed in an in vitro setting, and they may have altered relevance in a clinical situation, wherein factors like differences in tea varieties, amounts consumed over varying time periods, oral hygiene practices and other dietary supplements can be associated with the accumulation of extrinsic staining [44]. Although this study did not support the wound healing stimulation of guava extract, as reported in earlier animal studies [43], other research designs may yet show a benefit in its use in this regard. The analysis presented above emphasizes the need for a more nuanced framework of in vitro experiments to understand more fully the microbiological and eukaryotic cell response to guava extract mouth rinse.

## 5. Conclusions

In summary, the nutraceutical product guava was indigenously formulated into a mouth rinse utilizing an extract of its leaves, and various parameters were tested for its effectiveness. The observations of the current work showed that the formulation tested fulfilled the criteria of an oral care product. It has the ability to reduce biofilm formation and has displayed less staining potential as compared to CHX. Hence, this study corroborates earlier findings that guava mouth rinse may be considered a useful oral health care agent, especially for long-term use where CHX mouth rinses are contraindicated. In addition, our results have sustained the hypothesis that guava attenuates the inflammation in gingival epithelial keratinocytes treated with LPS by downregulating inflammatory markers. The results of this study show that guava may be considered a potent anti-inflammatory agent with a potential for oral therapeutic use as a mouth rinse against inflammation and can be added to the list of phytotherapeutic alternatives for healthy gingiva. Nevertheless, further clinical trials should be conducted over an extended time period to substantiate its long-term effects.

## Figures and Tables

**Table 1:** Mean (µg/mL) and percentage of guava and Chlorhexidine (CHX) released from teeth coated with 0.2% CHX mouth rinse and 0.15% guava mouth rinse. The student’s t-test analysis displayed that the percent release of 0.15% guava mouth rinse at 360 min was higher, which indicated enhanced retentive ability compared to 0.2% CHX mouth rinse.

	Group 1—0.15 % Guava Mouth Rinse			Group 2—0.2% CHX Mouth Rinse		
Time (min)	5	30	360	5	30	360
Mean ± SD in µg/mL	8.14 ± 2.41	7.010 ± 3.11	5.87 ± 4.41	177.24 ± 62.07	137.62 ± 54.89	124.82 ± 39.15
% Release	29.5%	16.11%	19.4%	57%	22.5%	10.25%

**Table 2:** Mean ± SD colony-forming units after exposure to the experimental and control agents. The Kruskal–Wallis test indicated a reduction in microbial colonies by four log steps for both 0.2% CHX and 0.15% guava mouth rinses (P < 0.05).

	Group 1 0.15% Guava Mouth Rinse (CFU/mL)	Group 2 0.2% CHX Mouth Rinse (CFU/mL)	Group 3 Distilled Water (CFU/mL)
Baseline	1 × 10^5^	1 × 10^5^	1 × 10^5^
24 h	2.03 × 10^2^ ± 0.81 × 10^2^	2.8 × 10^1^ ± 0.14 × 10^1^	9.05 × 10^8^ ± 2.48 × 10^10^
72 h	1.5 × 10^1^ ± 0.15 × 10^1^	0.5 × 10^1^ ± 0.1 × 10^1^	1.23 × 10^9^ ± 2.24 × 10^11^

**Table 3:** Mean changes in luminosity (Δ L*), red-green axis (Δ a*), yellow-blue axis (Δ b*), and overall color (Δ E*). The darkening (∆L*) and discoloration (∆E*) were recorded on all specimens. The enamel samples did not display substantial staining. Dentine samples showed significant staining by all the three test agents. Composite substrates, CHX, and distilled water, exhibited significant staining. Identical uppercase alphabets (to be read horizontally) indicate a significant difference.

	-	Guava	Chlorhexidine	Distilled Water
Enamel	ΔL	−10.43 ± 2.17	−18.55 ± 6.52	−8.85 ± 4.16
	Δa	4.36 ± 0.23 ^B^	9.82 ± 4.57 ^C^	2.30 ± 0.33 ^B,C^
	Δb	12.02 ± 0.98 ^B^	18.82 ± 8.75 ^C^	4.39 ± 4.70 ^B,C^
	ΔE	13.50 ± 1.64	24.27 ± 9.55	9.11 ± 4.21
Dentin	ΔL	−10.04 ± 1.12 ^A^	−31.42 ± 5.38 ^A,C^	−11.69 ± 3.07 ^C^
	Δa	7.11 ± 1.02 ^A,B^	18.03 ± 2.28 ^A,C^	2.03 ± 0.24 ^B,C^
	Δb	15.91 ± 1.91 ^A,B^	26.92 ± 2.60 ^A,C^	4.86 ± 0.73 ^B,C^
	ΔE	16.07 ± 1.56 ^A^	43.15 ± 6.79 ^A,C^	12.58 ± 3.12 ^C^
Composite	ΔL	2.06 ± 2.22	−3.66 ± 4.78 ^C^	6.42 ± 3.26 ^C^
	Δa	3.80 ± 1.96 ^A,B^	6.64 ± 0.65 ^A,C^	1.04 ± 0.35 ^B,C^
	Δb	7.40 ± 2.99	10.32 ± 1.14 ^C^	3.70 ± 0.49 ^C^
	ΔE	−1.088 ± 1.46	5.21 ± 4.8 ^C^	−6.21 ± 3.19 ^C^
